# Diagnostic test strategies in children at increased risk of inflammatory bowel disease in primary care

**DOI:** 10.1371/journal.pone.0189111

**Published:** 2017-12-06

**Authors:** Gea A. Holtman, Yvonne Lisman-van Leeuwen, Boudewijn J. Kollen, Obbe F. Norbruis, Johanna C. Escher, Laurence C. Walhout, Angelika Kindermann, Yolanda B. de Rijke, Patrick F. van Rheenen, Marjolein Y. Berger

**Affiliations:** 1 Department of General Practice, University of Groningen, University Medical Center Groningen, Groningen, the Netherlands; 2 Department of Pediatrics, Isala Hospital, Zwolle, The Netherlands; 3 Department of Pediatric Gastroenterology, Erasmus MC-Sophia Children’s Hospital, Rotterdam, the Netherlands; 4 Department of Pediatric Gastroenterology, Emma Children’s Hospital ⁄ Academic Medical Center, Amsterdam, the Netherlands; 5 Department of Clinical Chemistry, Erasmus MC, University Medical Centre, Rotterdam, The Netherlands; 6 Department of Pediatric Gastroenterology, University of Groningen, University Medical Centre Groningen, Groningen, the Netherlands; University of Nevada School of Medicine, UNITED STATES

## Abstract

**Background:**

In children with symptoms suggestive of inflammatory bowel disease (IBD) who present in primary care, the optimal test strategy for identifying those who require specialist care is unclear. We evaluated the following three test strategies to determine which was optimal for referring children with suspected IBD to specialist care: 1) alarm symptoms alone, 2) alarm symptoms plus c-reactive protein, and 3) alarm symptoms plus fecal calprotectin.

**Methods:**

A prospective cohort study was conducted, including children with chronic gastrointestinal symptoms referred to pediatric gastroenterology. Outcome was defined as IBD confirmed by endoscopy, or IBD ruled out by either endoscopy or unremarkable clinical 12 month follow-up with no indication for endoscopy. Test strategy probabilities were generated by logistic regression analyses and compared by area under the receiver operating characteristic curves (AUC) and decision curves.

**Results:**

We included 90 children, of whom 17 (19%) had IBD (n = 65 from primary care physicians, n = 25 from general pediatricians). Adding fecal calprotectin to alarm symptoms increased the AUC significantly from 0.80 (0.67–0.92) to 0.97 (0.93–1.00), but adding c-reactive protein to alarm symptoms did not increase the AUC significantly (p > 0.05). Decision curves confirmed these patterns, showing that alarm symptoms combined with fecal calprotectin produced the diagnostic test strategy with the highest net benefit at reasonable threshold probabilities.

**Conclusion:**

In primary care, when children are identified as being at high risk for IBD, adding fecal calprotectin testing to alarm symptoms was the optimal strategy for improving risk stratification.

## Introduction

Abdominal pain is a common gastrointestinal symptom in children that prompts a visit to the general practitioner [1–3]. In most children, abdominal symptoms are attributed to functional gastrointestinal disorders (FGIDs), and in a few children to an organic disease [4–6]. This distinction is important because children with FGIDs have no structural or biochemical abnormalities and can be managed in primary care [7,8]; moreover, excessive testing can sustain complaints and decrease patient well-being [9,10]. However, a thorough differential diagnosis is necessary to avoid delaying diagnosis and appropriate treatment of serious organic disease, such as inflammatory bowel disease (IBD) [11].

Differentiation between FGID and IBD is difficult, however, because symptoms are non-specific and frequently overlap. The absence of alarm symptoms (e.g., weight loss and rectal blood loss) may help general practitioners to exclude IBD and prevent the unnecessary referral of children with FGIDs [12]. However, these alarm symptoms are common and are often related to illnesses that could be safely managed in primary care (e.g., rectal bleeding caused by anal fissuring related to constipation). The trade-off between the benefits (early recognition of IBD) and harms (referral of FGIDs) of referral and the lack of adequate tools to discriminate children with IBD, triggers general practitioners to either refer children with abdominal symptoms for further diagnostic work-up or perform non-valid tests [4].

In primary care, evaluating alarm symptoms and blood markers is the most commonly used diagnostic strategy for triaging children with chronic gastrointestinal symptoms before referral to specialist care [13]. C-reactive protein has shown the best diagnostic performance of blood markers, but evaluation of its diagnostic value is limited to highly selected children [14,15]. More recently, fecal calprotectin has been shown to be a useful, simple, and non-invasive test that can exclude IBD in primary care [16]. Although test characteristics such as sensitivity, specificity, and area under the receiver operating characteristic curve (AUC) have been presented, the added value of c-reactive protein, or fecal calprotectin to alarm symptoms are unknown [17]. In conclusion, there is little evidence to recommend additional testing in children with chronic gastrointestinal symptoms in primary care.

In this study, we aim to determine the optimal diagnostic test strategy supporting decisions for referral to specialist care in children with suspected IBD by evaluating the added diagnostic performance of c-reactive protein and fecal calprotectin beyond alarm symptoms. For that purpose, we compared three test strategies: 1) alarm symptoms alone, 2) alarm symptoms plus c-reactive protein, and 3) alarm symptoms plus fecal calprotectin.

## Materials and methods

### Setting and participants

We conducted a prospective cohort study between July 2011 and September 2014 in the Netherlands. All children were followed for 12 months. We recruited 2 cohorts of children with chronic gastrointestinal symptoms: 1) children initially seen in one out of 38 participating general practices (primary care cohort) and 2) children initially referred by either a general practitioner or general pediatrician to one of the participating medical centers (4 general hospitals and 3 academic centers) (referred cohort). Children included in the primary care cohort were also included in the referred cohort when they had ≥1 alarm symptom or an abnormal blood test (Table 1), in accordance with the study protocol [18]. For this study we only included children in the referred cohort.

**Table 1:** Diagnostic criteria for alarm symptoms, blood markers, and fecal calprotectin.

Symptoms or tests	Measurement	Positive
**Alarm symptoms**		
Rectal blood loss	History	Rectal bleeding with defecation without constipation according to ROME III criteria
Family history of IBD	History	Affected first-degree relatives
Involuntary weight loss	History and physical examination	Involuntary decrease in weight of > 1 kg
Growth failure	History and physical examination	Target height range more than −1 standard deviation score
Extra-intestinal symptoms	Physical examination	Eyes (episcleritis, scleritis, uveitis), skin (erythema nodosum, pyoderma gangrenosum, psoriasis), mouth ulcers, finger clubbing, arthritis
Peri-anal lesions	Physical examination	Skin tags, hemorrhoids, fissures, fistulas, and/or abscesses
**Blood markers**		
Hemoglobin	Local laboratory	4–12 years < 7.1 mmol/l, boys 12–18 years < 8.1 mmol/l, girls 12–18 years < 7.4 mmol/l [22]
C-reactive protein	Local laboratory	> 10 mg/l [23]
Erythrocyte sedimentation rate	Local laboratory	> 20 mm/h [23]
platelet count	Local laboratory	> 450 × 10^9^/l [24]
**Fecal marker**		
Fecal calprotectin	ELISA (Phical test)	> 50 μg/g

Abbreviations: ELISA: enzyme-linked immunosorbent assay; IBD: inflammatory bowel disease.

For inclusion, children had to be aged 4–18 years and presenting with chronic diarrhea (soft or watery stool, matching scores 5–7 of the Bristol Stool chart, for ≥2 weeks or ≥2 episodes in the past 6 months) [19] or recurrent abdominal pain or discomfort (≥2 episodes in the past 6 months) [12,13]. An episode was defined as symptoms during three days or more. Participants were excluded if they had a known diagnosis of chronic organic gastrointestinal disease, had undergone endoscopic evaluation or fecal calprotectin measurement within the preceding 6 months, or had cognitive impairment or language problems that caused difficulty in understanding questionnaires. Furthermore, we excluded children with chronic use (>3 months) of antibiotics, non-steroid anti-inflammatory drugs (NSAID), or oral corticosteroids, as well as children aged under 4 years, because the calprotectin levels of these groups have been shown to be higher than those observed in healthy older children and adults [20,21].

The Medical Ethics Committee of the University Medical Centre Groningen, the Netherlands, approved the study. Parents of all children provided written informed consent, as did children aged 12 years or older.

### Baseline measurements

At baseline, the general practitioner or pediatrician performed a structured physical examination to identify the six alarm symptoms, using a standardized form [12,13]. A blood sample was taken from all patients to measure four blood markers at local laboratories (c-reactive protein, hemoglobin, erythrocyte sedimentation rate, and platelet count). Directly after baseline, all children collected a stool sample that was sent to a laboratory for storage at –80°C. At the end of the data collection period (September 2014), calprotectin was measured in the department of clinical chemistry at Erasmus MC Rotterdam, using a commercially available quantitative enzyme-linked immunosorbent assay (Phical test, CALPRO AS, Oslo, Norway). Alarm symptoms and the thresholds for blood markers and fecal calprotectin are presented in Table 1. All physicians, researchers, and patients were blinded to the outcome of the fecal calprotectin test, but not to the results of alarm symptoms or blood analyses. The technicians in all laboratories were blinded to the clinical characteristics and diagnoses of patients.

### Patient flow

After baseline assessment, a pediatric gastroenterologist decided whether to perform an endoscopy or not, based on interpretation of baseline findings and examination of the child. All children were followed for 12 months, and every 3 months a symptom questionnaire was completed by parent or child (if aged ≥10 years). The symptom questionnaire was developed in cooperation with experienced pediatric gastroenterologists and general practitioners (S1 Text. Children’s symptom questionnaire). At 12 months, either the general practitioner or pediatrician performed a structured physical examination of all children who still had chronic gastrointestinal symptoms and who had not received a diagnosis of IBD during the study. Children with at least one alarm symptom at this examination were seen again by the pediatric gastroenterologist who evaluated the need for endoscopy. If a child was lost to follow-up, the general practitioner or pediatrician was contacted to provide updated information on relevant diagnoses (IBD or other organic diseases) during the 12 months after baseline.

### Outcome

The diagnosis of IBD was confirmed by esophagogastroduodenoscopy and ileocolonoscopy, with histology, according to the Porto criteria [12]. Absence of IBD was defined as no endoscopic and histopathologic evidence of IBD or no indication for endoscopy within or at 12 months’ follow-up. Endoscopy was not considered to be indicated if there were no alarm symptoms for IBD or if the pediatric gastroenterologist considered that the identified alarm symptoms were not related to IBD.

### Statistical analyses

We calculated the test characteristics of alarm symptoms, blood markers and fecal calprotectin with 95% confidence intervals (CIs). In addition, we calculated the AUC for blood markers and fecal calprotectin.

We used logistic regression analysis to construct a basic model predicting the presence of IBD. The dependent variable was the IBD diagnosis (dichotomous). For the independent variable, we totaled the alarm symptoms into one variable (continuous) to comply with the number of events per variable (EPV) rule for obtaining adequate statistical power [25,26]. We added weighting scores to the alarm symptoms, because the symptoms are known to have different predictive values [12,13].

To evaluate the added value of c-reactive protein and fecal calprotectin (both continuous scaled) to alarm symptoms alone (basic model), the variables were added to the basic model and their probabilities were compared to those of the basic model by calculating differences between AUCs, using the method described by DeLong [27]. The models were also assessed with goodness-of-fit tests and calibration plots.

We used decision curve analysis to evaluate the clinical usefulness of decision-making based on the three diagnostic strategies [28–30]. We chose a range of threshold probabilities with an upper limit of 40%, because it is unrealistic that any general practitioner would accept more than a 40% risk of IBD for a referral to be justified [17]. Strategies with the highest net benefit at a particular threshold were considered preferable to alternative strategies.

To evaluate the verification pattern of which children received endoscopy at baseline, we compared baseline characteristics and risk of IBD in children with and without endoscopy at baseline. We expected that especially children at high risk would be subjected to an endoscopy.

If the distribution between variables in patients with and without missing data was different, the data were considered missing at random (MAR) [31]. In that case, we conducted multiple imputations (fully conditional specification, predictive mean matching, [32] 20 iterations, 20 datasets), using patient characteristics, symptoms, tests and outcome as predictors [33]. We used Rubin’s rule to calculate the pooled AUC [34]. We presented results of the non-imputed (complete case analysis) and imputed dataset. Statistical analyses were performed with IBM SPSS, Version 24 (IBM corp., Armonk, New York, USA) and STATA/SE 13 (STATA Corp, College station, TX, USA).

## Results

### Participants

**Fig 1:**
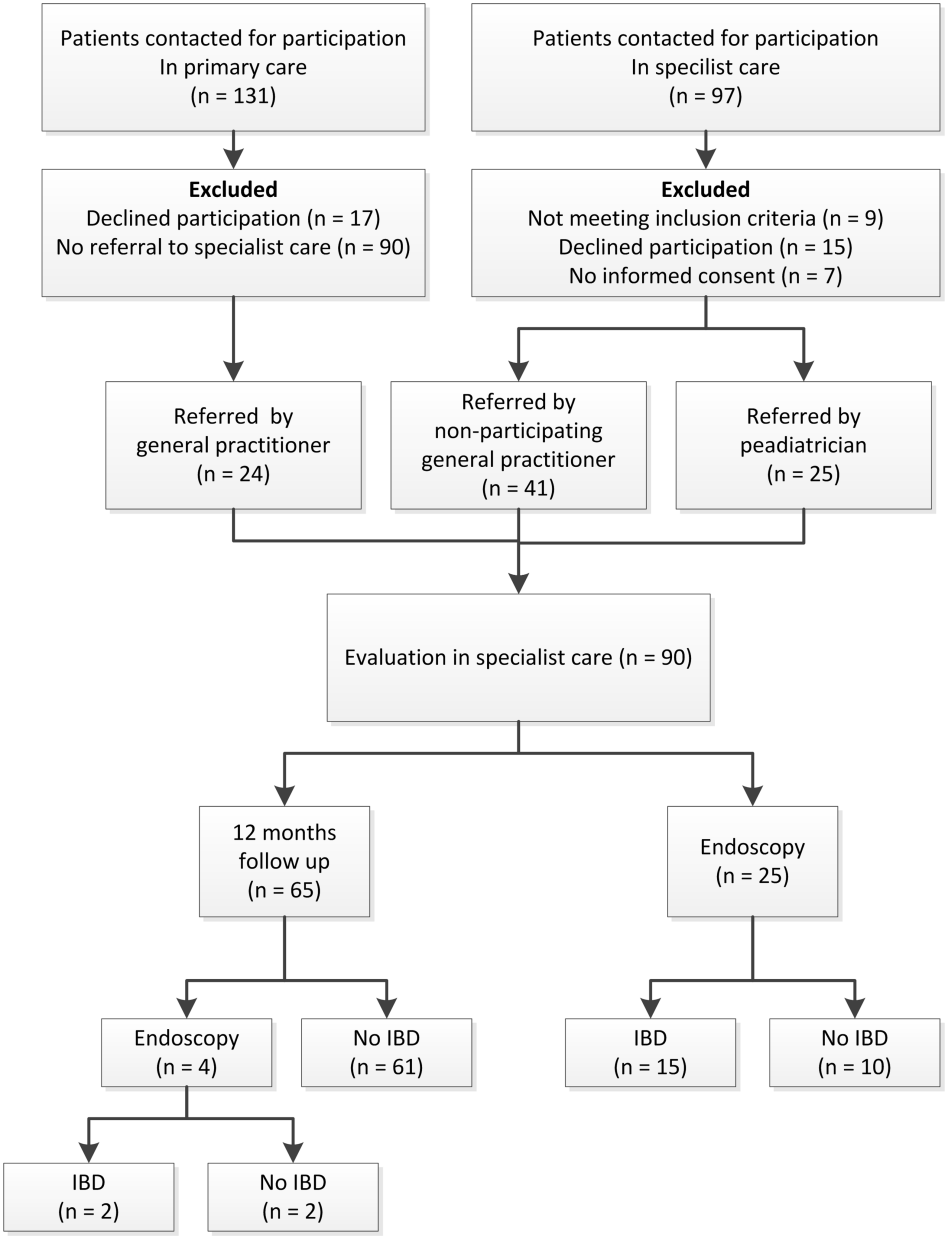
Patient flow diagram. Abbreviations: IBD: inflammatory bowel disease.

Fig 1 summarizes the patient flow in this study. Children referred to the pediatric gastroenterologist by a general pediatrician (n = 25) were older, more frequently had alarm symptoms, and were more likely to have IBD compared with children who were referred by a general practitioner (n = 65). Children who were subject to endoscopy at baseline (n = 25) more frequently had alarm symptoms, positive laboratory markers, and a high risk for IBD compared with children who underwent no endoscopy at baseline (n = 65) (Table 2).

**Table 2:** Baseline characteristics of children by referrer and whether endoscopy was performed at baseline.

	Referred by general practitioners (n = 65)	Referred by general pediatrician (n = 25)	No endoscopy At baseline (n = 65)	Endoscopy At baseline (n = 25)
Male sex (n (%))	29 (45)	8 (32)	27 (42)	10 (40)
Age in years at baseline (median, IQR)	10 (7–14)	14 (10–15.5)	9 (6–14)	15 (12–16)
**Duration symptoms (n (%))**				
<0.5 year	14 (22)	6 (24)	12 (19)	8 (32)
>1 year	41 (63)	9 (36)	42 (65)	8 (32)
**History and physical examination (n (%))**				
Growth failure	6 (9.2)	0 (0)	5 (8)	1 (4)
Involuntary weight loss	10 (15)	13 (52)	10 (15)	13 (52)
Rectal blood loss	13 (20)	14 (56)	16 (25)	11 (44)
Positive family history of IBD	9/64 (14)	2 (8)	6/64 (9)	5 (20)
Extra-intestinal symptoms	4 (6)	9 (36)	6 (9)	7 (28)
Peri-anal lesions	9 (14)	4/24 (17)	7 (11)	6/24 (25)
≥1 alarm symptoms	38 (59)	24 (96)	39 (60)	23 (92)
**Blood markers (n (%))**				
hemoglobin (cut-off is age/sex specific^a^)	5/61 (8)	6/24 (25)	4/61 (7)	7/24 (29)
C-reactive protein (>10 mg/l)	5/56 (9)	5/19 (26)	2/53 (4)	8/22 (36)
erythrocyte sedimentation rate (>20 mm/h)	8/59 (14)	8/24 (33)	4/59 (7)	12/24 (50)
Platelet count (>450 x10^9^/l)	4/61 (7)	3/24 (13)	4/61 (7)	3/24 (13)
≥1 blood marker	14/53 (28)	10/19 (53)	11/50 (22)	13/22 (59)
**Fecal test (n (%))**				
fecal calprotectin (>50 μg/g)	14/63 (22)	13/22 (59)	9/63 (14)	18/22 (82)
**Reference standard (n (%))**				
endoscopy	9 (14)	20 (80)	4 (6)	25 (100)
**Diagnoses (n (%))**				
IBD	5 (8)	12 (48)	2 (3)	15 (60)
FGIDs^b^	55 (85)	11 (44)	58 (89)	8 (32)

^a^ 4–12 years < 7.1 mmol/l, boys 12–18 years < 8.1 mmol/l, girls 12–18 years < 7.4 mmol/l.

^b^ Diagnosis of FGID was reached when after 12 months there were no signs of any inflammatory, anatomic, metabolic, or neoplastic pathology after thorough history, physical examination, and additional testing by the treating physician.

Abbreviations: FGID: functional gastrointestinal disorder; IBD: inflammatory bowel disease.

### Diagnoses

IBD was confirmed in 17 patients (19%), of whom 7 had Crohn’s disease, 8 had ulcerative colitis, and 2 had unclassified IBD. Of the 72 children (80%) with other diagnoses, 66 (73%) had a FGID, 3 (3%) had gastroenteritis (*salmonella enteric* [n = 2]; *Giardia lamblia* [n = 1]), 1 (1%) had reflux esophagitis, 1 (1%) had celiac disease, and 1 (1%) had a solitary rectal ulcer. In one patient, the diagnosis was not known because the child, who was aged 16 years, refused endoscopic evaluation at baseline and evaluation by a general practitioner at 12 months.

### Test characteristics

All tests were performed before endoscopy. The median time intervals between blood sampling and endoscopy were 34 and 69 days for children with and without IBD, respectively. The median time interval between stool sampling and endoscopy was 4 days for children with IBD and was 8 days for children without IBD. Of the 29 children who underwent endoscopy, 11 (2 missing) had a delay of more than one month. Overall, 25 children had missing data for one or more variable of interest. The children with and without missing data were comparable in all baseline characteristics, except for extra-intestinal symptoms and setting (S1 Table). To reduce the probability of selection bias, we imputed the missing data [31]. The results of the non-imputed and imputed dataset were similar (Table 3), therefore bias is less probable and we presented the results of the non-imputed dataset in the text.

**Table 3:** The various diagnostic models for IBD with the non-imputed and imputed datasets.

	Non-imputed DOR (95% CI)	Imputed Pooled DOR (95% CI)	Non-imputed AUC (95% CI)	Imputed Pooled AUC (95% CI)
Alarm symptoms	1.02 (1.01–1.04)	1.02 (1.01–1.04)	0.80 (0.67–0.92)	0.80 (0.69–0.90)
Alarm symptoms + c-reactive protein	1.02 (1.01–1.04)	1.02 (1.01–1.03)	0.88(0.78–0.98)	0.85(0.76–0.93)
	1.18 (1.04–1.33)	1.14 (1.01–1.27)		
Alarm symptoms + fecal calprotectin	1.01 (0.99–1.04)	1.02 (0.99–1.04)	0.97(0.93–1.00)	0.97(0.93–1.00)
	1.01 (1.003–1.02)	1.01 (1.003–1.03)		

Basic model consisted of the number of weighted alarm symptoms (Total score: 357): growth failure (weight: 51), involuntary weight loss (weight: 44), rectal blood loss (weight: 60), family history of IBD (weight: 53), extra-intestinal symptoms (weight: 78), peri-anal lesions (weight: 71). The mean weighting scores for the alarm symptoms were based on the independent opinions of 85 physicians who treat children with chronic gastrointestinal symptoms in different clinical settings. The physicians weighted the alarm symptoms using a visual analog scale from 0 (completely excludes that the child has IBD) to 100 (absolutely confirms that the child has IBD). Interpretation DOR: one point increase on a continuous scaled test result (weighted alarm symptoms, c-reactive protein, fecal calprotectin) increases the risk of IBD with the DOR-value. Abbreviations: DOR: Diagnostic Odds Ratio; AUC: area under the curve.

The diagnostic characteristics of alarm symptoms, blood markers, and fecal calprotectin are shown in S2 Table. The AUC of fecal calprotectin was 0.98 (0.96–1.00), which was significantly higher than that for all blood markers individually (p < 0.05).

### Added value of C-reactive protein and fecal calprotectin

The AUC of the basic model was 0.80 (0.67–0.92). Adding c-reactive protein or fecal calprotectin to the model increased the AUC to 0.88 (0.78–0.98) and 0.97 (0.93–1.00), respectively. However, this increase was only significant for fecal calprotectin (p < 0.05) (Table 3). The goodness-of-fit test was good for all models (Hosmer and Lemeshow test, p > 0.05). The calibration plots of all models displayed linear concordance between the observed and predicted probabilities of IBD.

### Decision curve analysis

**Fig 2:**
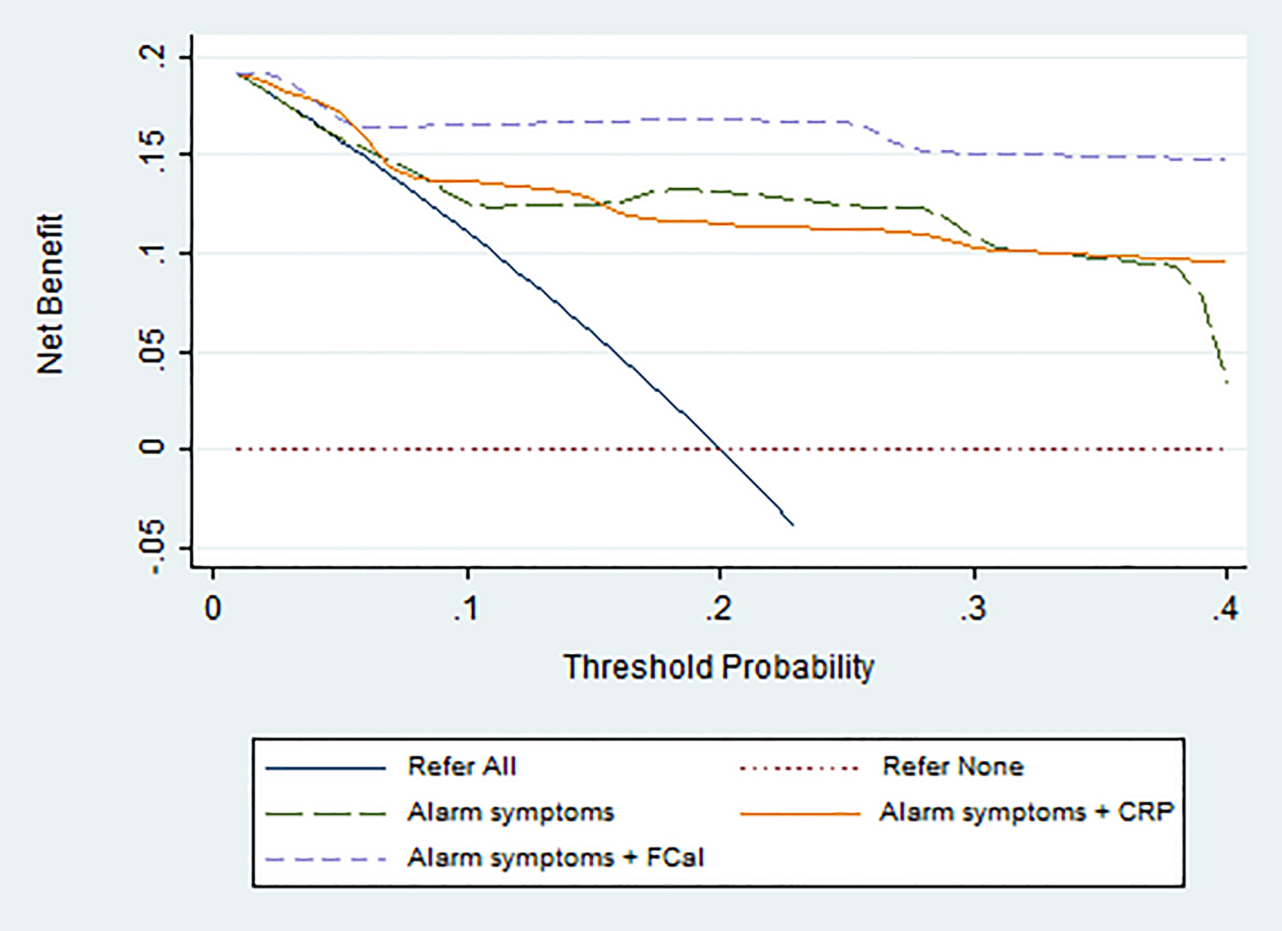
Decision curve for the three models predicting the outcome of IBD in the non-imputed dataset. Representative interpretation of the decision curve: the purple line representing the alarm symptoms + fecal calprotectin strategy shows a net benefit of 0.16 at a threshold probability of 20%. In this instance, a threshold probability of 20% means that a general practitioner would be willing to refer 5 children to prevent a delay in diagnosis for 1 child with IBD. The net benefit of 0.16 means that this strategy would lead to the referral of 160 per 1000 children at risk, with all referrals having IBD. Abbreviations: CRP: C-reactive protein, FCal: fecal calprotectin.

The decision curve analysis indicates that all three diagnostic strategies had higher net benefit at diagnostic threshold probabilities >5% when compared with the alternative of referring all children (Fig 2). Alarm symptoms in combination with fecal calprotectin had the highest net benefit at threshold probabilities between 5% and 40%. Table 4 shows that the test strategy of alarm symptoms plus fecal calprotectin generated a higher reduction in the numbers of referrals without missing a child with IBD at different threshold probabilities compared to all other strategies.

**Table 4:** Reduction in referral rate for further diagnostic work-up per 100 children using different threshold probabilities for the three test strategies.

Threshold probability	Alarm symptoms	Alarm symptoms + c-reactive protein	Alarm symptoms + fecal calprotectin
10%	–	15	32
15%	12	33	49
20%	35	40	59

Note: The reduction in the number of referral for further diagnostic work-up per 100 children without missing a child with IBD was calculated as follows: (net benefit strategy—net benefit of refer all) / [P_t_ / (1 –P_t_)] × 100.

## Discussion

In this study, 73% of children referred for evaluation of chronic gastrointestinal symptoms had FGIDs that could be managed in primary care, and the prevalence of IBD was 19%. Interestingly, for children in whom general practitioners considered a referral for further diagnostic work-up, c-reactive protein provided no additional diagnostic value when used in combination with alarm symptoms. By contrast, the addition of fecal calprotectin to alarm symptoms significantly improved the AUC. Compared to the strategy of adding c-reactive protein to alarm symptoms, adding fecal calprotectin to alarm symptoms showed the highest net benefit and produced the greatest reduction in the number of referrals for IBD without any false negatives.

In clinical practice, physicians use diagnostic threshold probabilities to determine when they need to initiate further testing before deciding on further management. The decision curve analysis in this study indicated, that in children with a threshold probability of IBD that was < 5%, testing with c-reactive protein or fecal calprotectin had limited additional value. Therefore, we advise against testing c-reactive protein or fecal calprotectin in children with a very low risk for IBD (e.g. without alarm symptoms). At threshold probabilities between 5% and 40%, however, the test strategy with alarm symptoms and fecal calprotectin showed highest net benefit. Moreover, the discriminative power of fecal calprotectin proved to be superior to all blood markers when compared individually. Therefore, on the bases of our results, we conclude that fecal calprotectin is the best laboratory test for use when seeking to further stratify children identified as at risk for IBD by history and physical examination. In our previous study in primary care, we showed that a calprotectin value below 50 μg/g feces can safely exclude IBD [16]. Therefore, a normal calprotectin value is a very reassuring test result that complements a thorough history and physical examination in children with chronic gastrointestinal symptoms. It is important that the GP is aware of factors that increase the fecal calprotectin value: the calprotectin value is increased in children with bacterial gastroenteritis, younger than four years, or using NSAIDs or antibiotics [21].

There are few publications about the added value for fecal calprotectin in children with symptoms suggestive of IBD. A study evaluating the added value of fecal calprotectin using the “clinical eye” of the pediatrician showed that fecal calprotectin reduced the need for referral to a pediatric gastroenterologist, with only a low risk of missing a child with IBD [23]. However, we could not determine how this approach incorporated alarm symptoms and blood markers. Other researchers constructed a model to predict the risk of having IBD based on fecal calprotectin and age,[35] and correctly identified 85.5% of children with a sensitivity of 0.81 and specificity of 0.92 (AUC 0.92). However, important predictors, such as alarm symptoms and blood markers, were not included in their model.

It is important to realize that our study included patients referred by general practitioners and general pediatricians. Although it was assumed that these represent the same patient population, the children referred by a pediatrician were more likely to have IBD, were older, and had more alarm symptoms compared with children referred by their general practitioner. This result is to be expected in the Dutch healthcare system, because pediatricians can only be consulted if patients are referred by a general practitioner, but pediatric gastroenterologists can be consulted if children are referred by either a general practitioner or a general pediatrician. Healthcare systems in the United Kingdom, Scandinavia, Canada, New Zealand, and Australia are similar [36].

A limitation of our study is that alarm symptoms are routinely assessed with blood markers in children with symptoms suggestive of IBD. Consequently, the reference standard was interpreted with prior knowledge of the test results of alarm symptoms and blood markers, which might have caused review bias and overestimation of the diagnostic accuracy of the alarm symptoms and blood markers [37]. Another limitation is that we did not perform endoscopies in children with a low likelihood of IBD, because this was considered unethical. There are two important aspects to consider when assessing whether the use of two reference standards lead to biased accuracy estimates: the verification pattern and the appropriateness of the follow-up [38]. Although the verification pattern was based on the clinical judgment of the pediatric gastroenterologist, and was thereby somewhat subjective, our results did show that children who received endoscopy at baseline were at higher risk for IBD than children who received follow-up. Therefore, if the test strategies were only evaluated in the children who received endoscopy at baseline, the results would probably be biased [39]. Although IBD is not a self-limiting disease and it is very rare for it to stay in remission for one year,[23] there is a very small chance that we missed a child with IBD; such data could alter the diagnostic values reported for the tests. However, follow-up is the best achievable option given the reality of clinical care [40].

Given that there were few children with IBD in our study (n = 17), we combined the weighted alarm symptoms into one variable based on the subjective opinions of 85 physicians. Despite this, the AUC of the combined alarm symptoms without weighting was comparable to that with weighting. We also evaluated the added value of two important markers based on eight events per variable; although it is recommend to have at least 10 events per variable it has been shown that 5 events per variable is appropriate [25]. A larger study with more events is needed for the development of a prediction model for IBD based on single alarm symptoms, blood markers, and fecal calprotectin results [29]. Moreover, an exploration of whether age influences the prediction model is needed.

## Conclusions

The addition of fecal calprotectin to an evaluation of alarm symptoms was the optimal strategy for stratifying children identified as being at risk of IBD by their general practitioner. This strategy is different to that published in several guidelines, which recommend testing for c-reactive protein and other blood markers of inflammation when alarm symptoms are present [13,41]. It should be noted that we focused on IBD alone in this study, whereas general practitioners must evaluate whether symptoms are related to any organic disease, including celiac disease. Further studies are therefore needed to investigate whether the new test strategy combining the assessment of alarm symptoms with testing for fecal calprotectin can improve decision-making in clinical practice. These studies should also include a cost–benefit analysis.

## Supporting information

S1 Text Children’s symptom questionnaire. (PDF)

S1 Table Complete data, number missing per variable, and difference in distribution between children with and without missing data. (DOCX)

S2 Table Diagnostic characteristics of alarm symptoms, blood markers, and fecal calprotectin for IBD with a pre-test probability of 19%, using the imputed dataset. (DOCX)
