# Supplementary material for: Diagnostic test strategies in children at increased risk of inflammatory bowel disease in primary care
Source: PLoS One. 2017 Dec 6;12(12):e0189111. doi: 10.1371/journal.pone.0189111 (PMC5718464; doi:10.1371/journal.pone.0189111)

CODE NR

THE CHILDREN'S SYMPTOMS QUESTIONNAIRE

(AGED 10 TO 18 YEARS)

DARM ONDERZOEK BIJ KINDEREN

Instructions

This questionnaire is about symptoms that you may have if you have been diagnosed with a chronic inflammatory bowel disease. Your answers will be kept strictly confidential. If you find it difficult to answer a question, please try to answer the question as best as you can. In some of the questions a number will be asked, please try to answer this as accurately as possible.

How to register your answer correctly:

If you want to change your answer, you cross the wrong answer (see example) and fill in the right answer.

General questions

1. I have filled in this questionnaire on ☐☐ ☐☐ ☐☐☐☐
day month year

2. I was born on ☐☐ ☐☐ ☐☐☐☐
day month year

3. I am a
☐0 boy
☐1 girl

Visit to GP or hospital (paediatrician)

The next questions are about visits to your GP, or paediatrician in the hospital, due to symptoms of diarrhoea or other gastro-intestinal symptoms.

4. **In the past 3 months**, have you ever seen your GP because you had loose stools? The GP visit at the start of this study does not count.
☐0 No
☐1 Yes

5. **In the past 3 months,** have you ever been to a paediatrician in the hospital because you had gastro-intestinal complaints?
☐0 No
☐1 Yes, to: Doctor:

Hospital:

Department:

6. **In the past 3 months,** have you ever had an intestinal research (endoscopy)?

☐0 No

☐1 Yes

7. **In the past 3 months,** have you been diagnosed with a gastro-intestinal disease? (Crohn's disease, ulcerative colitis, celiac disease)

☐0 No

☐1 Yes, namely Crohn's disease

☐2 Yes, namely ulcerative colitis

☐3 Yes, namely coeliac disease

Questions about your stool (poop)

The next questions deal with diarrhoea. We refer to the Bristol stool form scale. This stool form is attached to the questionnaire (appendix 1). On this form you see 7 pictures of how your stool might look like.

8. **In the past 3 months,** have you ever had stools that look like the numbers 5, 6, or 7 from the Bristol stool form scale?

☐0 No

☐1 Yes, namely number 5

☐2 Yes, namely number 6

☐3 Yes, namely number 7

9. For how long did you have stools like number 5, 6, or 7 of the Bristol stool form scale?

☐9 I had no stools like number 5, 6, or 7

☐0 Less than 1 week

☐1 Between 1 and 2 weeks

☐2 Between 2 and 4 weeks

☐3 4 or more weeks

10. How many times did you have stools like numbers 5, 6, or 7 of the Bristol stool form scale?

    - □9 I had no stools like number 5, 6, or 7 of the Bristol stool form scale
    - □0 Once
    - □1 2 or more times

11. **In the past 3 months,** have you ever had blood in your stool?

    - □0 No
    - □1 Yes

12. **In the past 3 months,** have you ever awoken because you had to poop?

    - □0 No
    - □1 Yes

Questions about abdominal pain

13. **In the past 3 months**, have you ever had abdominal pain or uncomfortable feeling in the abdomen?

    - □0 No
    - □1 Yes

14. **In the past 3 months**, how many times have you had abdominal pain?

    - □0 I have had no abdominal pain
    - □1 Once
    - □2 Twice
    - □3 3 or more times

15. For how long did you have an episode of abdominal pain?

    - □0 I had no abdominal pain
    - □1 Less than 1 day
    - □2 More than 1 day and less than 1 week
    - □3 More than 1 week

Questions about weight and length

We would like to have some information about your weight. Maybe your parents have a balance scale. We would ask you to weight yourself on this balance scale. It is important that you do this in the morning after you have waken up, and before you get dressed.

16. I weight at the moment: ☐ kg ☐9 I do not know

17. **In the past 3 months**, have you weight changed?
    - ☐0 No, my weight has not changed
    - ☐1 Yes, my weight has *decreased*, with ☐ kg ☐9 do not know how many kg
    - ☐2 Yes, my weight has *increased*, with ☐ kg ☐9 do not know how many kg

18. If your weight has *decreased*, have you bothered to lose weight or have you lost weight spontaneously?
    - ☐0 Not applicable, my weight has not decreased
    - ☐1 I have bothered to lose weight
    - ☐2 I have lost weight spontaneously

Now we ask you to measure your body length with the tape measure you received from us. Measure your body length, for example, together with one of your parents. It is important to do this without wearing your shoes.

19. My length is ☐ cm ☐9 I do not know

Other symptoms

20. **In the past 3 months**, have you suffered from thick, aching or red joints without having fallen or hit something? Examples of joints are a knees, elbows, or wrists.

- ☐0 No
- ☐1 Yes, namely in

21. **In the past 3 months**, have you had a lack of appetite?

- ☐0 No
- ☐1 Yes

22. **In the past 3 months**, have you ever shown signs of fatigue?

- ☐0 No
- ☐1 Yes

23. **In the past 3 months**, have you ever had a fever (higher than 37.5°C)?

- ☐0 No
- ☐1 Yes

24. **In the past 3 months**, have you ever had painful, red eyes? For instance due to an inflammation?

- ☐0 No
- ☐1 Yes

25. **In the past 3 months**, have you ever had spots or a rash on your skin?

- ☐0 No
- ☐1 Yes

26. **In the past 3 months**, have you ever had aphthous ulcers in your mouth?

- ☐0 No
- ☐1 Yes

Questions about your family

27. How many siblings do you have?

☐ Brothers ☐$_0$ I don't have any siblings

☐ Sisters

28. Was one of your family members ever diagnosed with a gastro-intestinal disease? (Crohn's disease, ulcerative colitits, coeliac disease)

Brother
- ☐$_0$ No
- ☐$_1$ Does not apply to me
- ☐$_2$ I do not know
- ☐$_3$ Yes, namely with ☐

Sister
- ☐$_0$ No
- ☐$_1$ Does not apply to me
- ☐$_2$ I do not know
- ☐$_3$ Yes, namely with ☐

Father
- ☐$_0$ No
- ☐$_2$ I do not know
- ☐$_3$ Yes, namely with ☐

Mother
- ☐$_0$ No
- ☐$_2$ I do not know
- ☐$_3$ Yes, namely with ☐

You might want to tell us something we have not asked you for in this questionnaire. If so, please write it down below. We are also pleased to hear from you if any of the questions were hard to answer and why this was so. In that case, do not forget to write down to which number of the question (and the page number) your point refers to!

This is the end of the questionnaire. Thank you so much for filling it in!

Finally, please look through all the questions to find any you have forgotten to answer!

APPENDIX 1. BRISTOL STOOL FORM SCALE

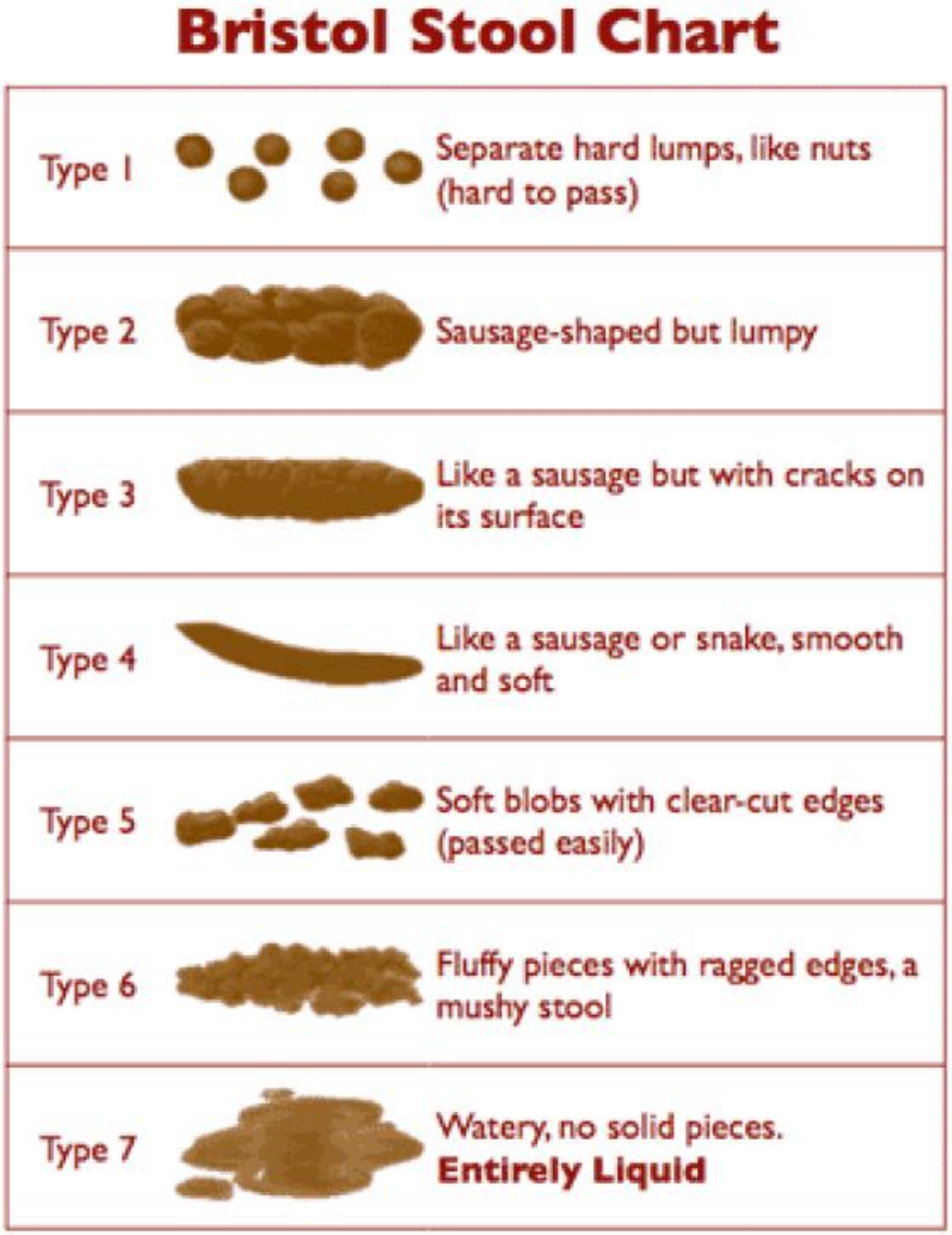

Supplement: S1 Text — (PDF) [file pone.0189111.s001.pdf]
