# Supplementary material for: Diagnostic test strategies in children at increased risk of inflammatory bowel disease in primary care
Source: PLoS One. 2017 Dec 6;12(12):e0189111. doi: 10.1371/journal.pone.0189111 (PMC5718464; doi:10.1371/journal.pone.0189111)
Supplement: S1 Table — (DOCX) [file pone.0189111.s002.docx]

**S1 Table.** **Complete data, number missing per variable, and difference in distribution between children with and without missing data**

|  | **Complete data**  **N = 65 (72%)** | **Missing**  **N (%)** | **P-value** |
| --- | --- | --- | --- |
| Male sex (n (%)) | 28 (43.1) | 0 | 0.8 |
| Age in years at baseline (median, IQR) | 11 (8-15) | 0 | 0.6 |
| IBD (n (%)) | 13 (20) | 1 (1.1) | 0.4 |
| **Setting** (n (%)) |  | 0 | 0.003* |
| Primary care | 22 (33.8) |  |  |
| Secondary care | 27 (41.5) |  |  |
| Tertiary care | 16 (24.6) |  |  |
| **Alarm symptoms** (n (%)) |  |  |  |
| Growth failure | 5 (7.7) | 0 | 1.0 |
| Involuntary weight loss | 16 (24.6) | 0 | 0.6 |
| Rectal blood loss | 21 (32.3) | 0 | 0.6 |
| Positive family history of IBD | 8 (12.3) | 1 (1.1) | 0.4 |
| Extra-intestinal symptoms | 7 (10.8) | 0 | 0.03* |
| Peri-anal lesions | 11 (16.9) | 1 (1.1) | 0.2 |
| **Blood markers** (median (IQR)) |  |  |  |
| hemoglobin (mmol/l) | 8 (7.7-8.4) | 5 (5.6) | 0.8 |
| C-reactive protein (mg/l) | 1 (1-3.6) | 15 (16.7) | 0.8 |
| erythrocyte sedimentation rate (mm/h) | 7 (4-13.5) | 8 (7.8) | 0.4 |
| Platelet count (x10^9^/l) | 299 (253-359) | 5 (5.6) | 0.6 |
| **Fecal test** (median (IQR)) |  |  |  |
| Fecal calprotectin (μg/g) | 22 (20-101) | 5 (5.6) | 0.6 |

*P < 0.05; Difference between children with and without missing data was tested with chi^2^ for categorical variables, Independent sample T-Test for continuous scaled normally distributed variables, Mann-Whitney test for continuous scaled non-normally distributed variables.
