# Supplementary material for: Diagnostic test strategies in children at increased risk of inflammatory bowel disease in primary care
Source: PLoS One. 2017 Dec 6;12(12):e0189111. doi: 10.1371/journal.pone.0189111 (PMC5718464; doi:10.1371/journal.pone.0189111)
Supplement: S2 Table — (DOCX) [file pone.0189111.s003.docx]

**S2 Table.** **Diagnostic characteristics of alarm symptoms, blood markers, and fecal calprotectin for IBD with a pre-test probability of 19%, using the imputed dataset**

|  | **Sens**  **(95% CI)** | **Spec**  **(95% CI)** | **PPV**  **(95% CI)** | **NPV**  **(95% CI)** | **LR+**  **(95% CI)** | **LR –**  **(95% CI)** | **DOR**  **(95% CI)** | **Area under ROC curve (95% CI)** |
| --- | --- | --- | --- | --- | --- | --- | --- | --- |
| **Alarm symptoms** | |  |  |  |  |  |  |  |
| Involuntary weight loss | 0.69  (0.45-0.85) | 0.85  (0.75-0.91) | 0.52  (0.33-0.71) | 0.92  (0.83-0.96) | 4.53  (2.41-8.50) | 0.37  (0.18-0.75) | 12.22  (3.70-40.40) | - |
| Rectal blood loss | 0.49  (0.28-0.70) | 0.74  (0.63-0.83) | 0.31  (0.17-0.50) | 0.86  (0.75-0.92) | 1.90  (1.01-3.53) | 0.69  (0.43-1.11) | 2.74  (0.94-8.04) | - |
| Positive family history of IBD | 0.11  (0.03-0.34) | 0.87  (0.78-0.93) | 0.18  (0.51-0.47) | 0.80  (0.70-0.88) | 0.91  (0.22-3.84) | 1.01  (0.84-1.22) | 0.90  (0.18-4.59) | - |
| Growth failure | 0.00  (0.00-0.18) | 0.92  (0.83-0.96) | 0.00  (0.00-0.39) | 0.79  (0.69-0.87) | 0.00  (-) | 1.09  (1.02-1.17) | 0.00  (-) | - |
| Extra-intestinal symptoms | 0.29  (0.13-0.52) | 0.89  (0.79-0.94) | 0.38  (0.18-0.65) | 0.84  (0.74-0.90) | 2.59  (0.97-6.96) | 0.80  (0.59-1.09) | 3.23  (0.91-11.51) | - |
| Peri-anal lesions | 0.39  (0.20-0.62) | 0.90  (0.81-0.95) | 0.50  (0.26-0.73) | 0.86  (0.77-0.92) | 4.09  (1.64-10.19) | 0.67  (0.46-0.99) | 6.10  (1.77-20.99) | - |
| **Blood markers** |  |  |  |  |  |  |  |  |
| C-reactive protein | 0.50  (0.29-0.71) | 0.95  (0.87-0.98) | 0.69  (0.42-0.87) | 0.89  (0.80-0.94) | 9.4  (3.2-27.3) | 0.53  (0.53-0.84) | 17.8  (4.46-71.20) | 0.79  (0.69-0.90) |
| erythrocyte sedimentation rate | 0.57  (0.35-0.77) | 0.91  (0.82-0.96) | 0.60  (0.37-0.79) | 0.90  (0.81-0.95) | 6.19  (2.71-14.17) | 0.47  (0.27-0.82) | 13.11  (3.81-45.14) | 0.80  (0.68-0.92) |
| Platelet count | 0.18  (0.07-0.41) | 0.94  (0.86-0.98) | 0.43  (0.16-0.74) | 0.83  (0.73-0.89) | 3.09  (0.80-11.90) | 0.87  (0.69-1.09) | 3.55  (0.75-16.83) | 0.70  (0.57-0.84) |
| hemoglobin | 0.43  (0.24-0.66) | 0.95  (0.88-0.98) | 0.68  (0.40-0.88) | 0.88  (0.78-0.93) | 9.01  (2.84-28.55) | 0.59  (0.39-0.90) | 15.16  (3.63-63.37) | 0.77  (0.65-0.88) |
| **Fecal marker** |  |  |  |  |  |  |  |  |
| fecal calprotectin | 0.99  (0.81-1.00) | 0.84  (0.74-0.91) | 0.60  (0.42-0.75) | 1.00  (0.94-1.00) | 6.17  (3.65-10.44) | 0.00  (0.00-3.33) | - | 0.98  (0.96-1.00) |

Cut-offs blood markers and fecal calprotectin: hemoglobin (4-12 years < 7.1 mmol/l, boys 12-18 years < 8.1 mmol/l, girls 12-18 years < 7.4 mmol/l), C-reactive protein ( > 10 mg/l), erythrocyte sedimentation rate ( > 20 mm/h), Platelet count ( > 450 x10^9^/l), fecal calprotectin (> 50 μg/g). Abbreviations: CI: Confidence Interval; Sens: sensitivity; Spec: Specificity; NPV: negative predictive value; PPV: positive predictive value; LR+: positive likelihood ratio; DOR: Diagnostic Odds Ratio; ROC: receiver-operator curve.
